# Low body mass index is associated with higher rates of mild cognitive impairment and worse memory performance among older Canadian adults: An analysis of the Canadian Longitudinal Study on Aging (CLSA) database

**DOI:** 10.1177/13872877261464179

**Published:** 2026-07-11

**Authors:** Nadine Akbar, Dewei Lin, Yan Hoi Caleb Cheung, Asha Mohamed, Mohammad Chowdhury, Sotirios Damouras

**Affiliations:** 1 Institute of Health Policy, Management and Evaluation, 7938 University of Toronto, Toronto, Ontario, Canada; 2 8534 Humber River Health, Research Institute, Toronto, Toronto, Ontario, Canada; 3 School of Rehabilitation Therapy, Faculty of Health Sciences, Faculty of Health Sciences, 4257 Queen's University, Kingston, Ontario, Canada; 4 Department of Biostatistics, Gillings School of Global Public Health, 2331 University of North Carolina at Chapel Hill, Chapel Hill, NC, USA; 5 Department of Computer and Mathematical Sciences, 33530 University of Toronto Scarborough, Toronto, Ontario, Canada; 6 Department of Psychiatry, 2129 University of Calgary, Calgary, Alberta, Canada; 7 Provincial Research Data Services, 3146 Alberta Health Services, Calgary, Alberta, Canada

**Keywords:** Alzheimer's disease, body mass index, CLSA, cognition, memory, mild cognitive impairment, obesity

## Abstract

**Background:**

Both low and high body mass index (BMI) have been identified as risk factors for dementia, though the association between the range of BMI classifications and presence of mild cognitive impairment (MCI) and memory performance warrants further investigation.

**Objective:**

This study aimed to investigate the association between the range of BMI classifications and presence of MCI, as well as overall memory performance, among a large Canadian sample of older adults.

**Methods:**

This study utilized data from 40,232 participants from the Canadian Longitudinal Study on Aging (CLSA). The association between BMI and cognitive function was examined at baseline and first follow-up (3 years). Linear regression was used to model the relationship between BMI and memory performance, while logistic regression was used to assess the association between BMI and the odds of meeting criteria for MCI.

**Results:**

In the final regression model, a weak (partial eta-squared=0.8%, 95% CI [0.63%, 0.97%]) inverted-U relationship between BMI and REYII memory performance was demonstrated, with normal BMI showing the best performance. In the final logistic regression model, being underweight was associated with 3.22 times greater odds (95% CI [1.63, 6.39]) of meeting criteria for MCI compared to normal BMI. No association was demonstrated between change in BMI and change in cognitive performance between baseline and 3-year follow-up.

**Conclusions:**

Being underweight is linked to higher odds of MCI, suggesting the importance of adequate nutrition in preserving cognitive health among older adults.

## Introduction

Considered a global epidemic, obesity impacts approximately 30% of Canadian adults^1^ with prevalence rates continuing to rise.^2^ Obesity is associated with greater risk of a number of chronic conditions such as hypertension, diabetes and depression.^3–5^ Obesity has also been shown to be a risk factor for Alzheimer's disease (AD) and related dementias, independent of vascular and diabetes comorbidities.^6,7^ Mild cognitive impairment (MCI), considered a transitional state between normal aging and dementia, has also been shown to be more common among moderately to severely obese older adults (up to 50%) compared to the general population (6%).^8^

Obesity is most commonly defined using body mass index (BMI). The relationship between the entire range of BMI classes (underweight, normal, overweight, obesity I, II and III) and cognitive function is complex and influenced by factors such as age, sex, and race. For example, obesity has been shown to be more negatively associated with cognition among women than men.^9,10^ Also, beyond age 75, evidence has been shown for the “obesity paradox” where higher BMI is more protective with regards to cognitive function, particularly for executive function.^11,12^ Low BMI/being underweight has also been shown to be a risk factor for dementia,^13^ especially among Asian older adults.^13,14^

The primary research question that this current study aims to answer is the nature of the relationship between classes of BMI and cognitive function among Canadian older adults using data from the Canadian Longitudinal Study on Aging (CLSA). The CLSA is a prospective cohort study of over 50,000 Canadian adults aged between 45 and 85 followed for at least 20 years.^15^ Being the first database of this kind in Canada, it offers a unique opportunity to examine the complex relationship between the range of BMI classifications and cognitive function among a large sample of Canadian older adults. The CLSA database also includes variables that can be used to classify participants according to indication of MCI, thus also allowing a unique opportunity to examine the relationship between BMI classification and presence of MCI. In order to answer the primary research question, this study had two main objectives. For Objective 1, we aimed to determine the cross-sectional relationship between BMI classification and (a) memory performance, and (b) indication of MCI, using baseline data from the CLSA. We hypothesized that there will be presence of an inverted U relationship with participants classified as underweight and obese (Class I, II and III) showing lower level of memory performance and higher rates of MCI compared to normal BMI.^16^ For Objective 2, we aimed to determine whether 3-year changes in BMI (from baseline to first follow-up) were associated with changes in memory performance. We hypothesized that 3-year decreases in BMI will be associated with improvements in cognitive function.^17^

## Methods

This study utilized data from both the Tracking (data collected remotely, using computer assisted telephone interviews) and Comprehensive (data collected in person using in-home interviews, physical assessments and biospecimen collection) cohorts of the CLSA. The CLSA is a prospective cohort study that utilized a combination of provincial healthcare registration databases and random digit dialing to sample from the Canadian population.^18,19^ For Objective 1, as we were interested in cross-sectional associations between BMI and cognitive performance, only the baseline data (collected from 2011 to 2015) were used. For Objective 2, both the baseline and follow-up 1 (from 2014–2018) data were used. Ethics approval for this study was obtained from Veritas IRB Inc (Application number 2900).

Exclusion criteria for the CLSA study include the following^18^: (a) unable to respond in either French or English, (b) residents of the three territories, (c) full-time members of the Canadian Armed Forces, (d) individuals living in long-term care institutions at the time of recruitment, (e) persons living on reserves and other Aboriginal settlements. Individuals who are cognitively impaired at the time of recruitment are also excluded as determined by not being able to understand the purpose of the study (i.e., unable to give informed consent) and/or provide reliable data. For our study, we also excluded data from participants who were pregnant and self-reported neurological conditions that would affect cognitive functioning including stroke or cerebrovascular accident, Parkinsonism, multiple sclerosis, epilepsy and/or dementia or Alzheimer's disease. Lastly, we excluded participants with missing data on any variables of interest in this study. Given the substantial sample size, we did not employ missing data imputation methods. Details on the number of excluded participants and reasons for exclusion are presented in Supplemental Table 1. The final samples had similar demographic characteristics as the entire survey data, and consisted of 40,232 participants for Objective 1 and 27,781 participants for Objective 2.

### Body mass index

The BMI classifications (international standard) reported within the CLSA include: underweight (BMI<18.5 kg/m^2^), normal (BMI of 18.5–24.89 kg/m^2^), overweight (BMI of 25.0–29.9 kg/m^2^), obesity class I (BMI of 30.0–34.9 kg/m^2^), obesity class II (BMI of 35.0–39.9 kg/m^2^), and obesity class III (BMI of 40.0 + kg/m^2^). For the Tracking cohort (telephone interviews), BMI was calculated based on self-reported height and weight. For the Comprehensive cohort (in-person physical assessment), BMI was calculated based on physical measures of height and weight. For Objective 2, change in BMI over time was calculated by subtracting the BMI at baseline from BMI at 3-year follow-up which was then categorized according to the categories of (1) BMI decrease more than 10%, (2) BMI decrease of 5–10%, (3) BMI change of less than 5%/ no change, (4) BMI increase of 5–10%, and (5) BMI increase of more than 10%.^20^

### Cognitive scores

Memory performance was indicated based on performance on the Rey Auditory Verbal Learning Test (RAVLT) delayed recall (REYII). Raw scores (not the converted, normative corrected scores) were entered into all models as this would allow for the entering of demographic variables associated with cognitive performance into the regression models (e.g., age, education, sex), as well their interactions with BMI. The RAVLT was selected as this measure has been shown to have high sensitivity and classification accuracy for detecting MCI and AD.^21^ For Objective 2, change in cognitive performance was categorized as (a) stable, (b) declined, or (c) improved based on REYII reliable change indicator, z-score change more than 1.645.^22^

### Mild cognitive impairment

MCI was defined as performance 1.5 standard deviations (SD) below the age- and sex-adjusted mean on 2 or more of the 4 cognitive tests administered as part of the CLSA (REYI measuring immediate memory recall, REYII measuring delayed memory recall, Animal Fluency measuring generative verbal fluency, Mental Alteration Test^23^ measuring speeded alternation of ascending letters and numbers), which has been used by the CLSA to define overall cognitive impairment,^22^ as well as preserved activities of daily living (no functional impairment or mild impairment based on the Older Americans’ Resources and Services [OARS] Multidimensional Functional Assessment Questionnaire^24^). The OARS scale measures abilities such as ability to dress, feed, take care of appearance, walk, get out of bed, take bath, incontinence, telephone, travel, shopping, prepare meals, do housework, take medicine, handle money according to classification of (a) without help, (b) with some help, and (c) completely unable. This definition of MCI has been used in another CLSA study of one of the study authors^25^ and MCI criteria according to evidence of cognitive impairment but preserved activities of daily living has been used to define MCI in other studies.^26^

### Other variables

In all regression models, either linear or logistic, we included a set of demographic and design-related variables as covariates to account for factors known to influence cognitive performance and to reflect the CLSA sampling structure. Sex (M versus F), age (4 categories of 45 to 55, <55 to 65, <65 to 75, <75 to 85), education level (4 categories of Low: less than secondary school graduation, Medium: secondary school graduation but no post-secondary education, High-lower: some post-secondary education, and High-upper: post-secondary degree/ diploma), and race (non-White versus White) were included in all models as potential confounders. With regards to race, the number of individuals that self-identified as non-White was very low compared to White which is why race was not further categorized. The non-White category included the following groups: Chinese, Japanese, Korean, South Asian, Black, Latin American, Arab, West Asian, Southeast Asian and Filipino. Geostrata defined as province (AB, BC, MB, NB, NL, NS, ON, PE, QC, SK) crossed with Data Collection Site (DCS) versus non-DCS regions, and Cohort (Comprehensive versus Tracking) were included to account for the complex CLSA sampling design. A summary of participants’ baseline demographic characteristics described through these variables is provided in Table 1. Note that the cohorts (Comprehensive versus Tracking) were not perfectly aligned with the DCS versus non-DCS designation in Geostrata values, since telephone interviews were also conducted within DCS boundaries. Main effects for age, sex, education, and geographic strata were included in all models by default, as recommended in the CLSA Technical Document^27^ because these variables were used in the construction and calibration of the weights, and form part of the survey design. Other variables and interaction terms were included in the models if they (or their higher order terms) were statistically significant.

**Table 1.** Other explanatory variables included in the models (beyond BMI classification) with corresponding levels and frequencies at baseline.

Variable	Category	Count	Percent (%)
Sex	F	20,527	51.0
	M	19,705	49.0
**Age**	[45,55]	12,342	30.7
	(55,65]	13,214	32.8
	(65,75]	8894	22.1
	(75,85]	5782	14.4
**Education**	Low	2584	6.4
	Medium	4369	10.9
	High-lower	3016	7.5
	High-upper	30,263	75.2
**Race**	White	38,634	96.0
	non-White	1598	4.0
**Geostrata**	ON _Ottawa	2911	7.2
	AB _Calgary	2508	6.2
	AB _Non_DCS	1282	3.2
	BC _Non_DCS	1113	2.8
	BC _SFU_UBC	3213	8.0
	BC _Victoria	2798	7.0
	MB _Manitoba DCS	3170	7.9
	MB _Non_DCS	622	1.5
	NB _Non_DCS	1011	2.5
	NL _Memorial	2230	5.5
	NL _Non_DCS	671	1.7
	NS _Dalhousie	3048	7.6
	NS _Non_DCS	852	2.1
	ON _Hamilton	2690	6.7
	ON _Non_DCS	3327	8.3
	PEI_Non_DCS	891	2.2
	QC _McGill	2570	6.4
	QC _Non_DCS	1489	3.7
	QC _Sherbrooke	2711	6.7
	SK _Non_DCS	1125	2.8
**Cohort**	Comprehensive	25,109	62.4
	Tracking	15,123	37.6

### Data analysis

Mean REYII scores and absolute MCI rates were calculated with inflation weights applied.^27^ For Objective 1a (association between BMI classification and REYII performance) linear regression analysis was used with the raw score on the REYII as the outcome measure. For Objective 1b (association between BMI classification and presence of MCI), logistic regression analysis was used with presence of MCI as the (binary) outcome. The presented linear and logistic regression models included as explanatory variables the BMI Classification, all survey design variables (sex, age group, education level, Geostrata), potential confounders (race and cohort), and any interactions thereof that were statistically significant at the 5% level. For Objective 2, chi-square tests were used to examine the association between individual change in BMI and individual change in cognitive performance over a 3-year period. All analyses were performed with the R Statistical Software (version 4.4.1),^28^ and both linear and logistic regression modelling was conducted using the “survey” package (version 4.4.8)^29^ to account for the complex survey design.

## Results

### Association between BMI classification and memory/ REYII performance

**Figure 1.**
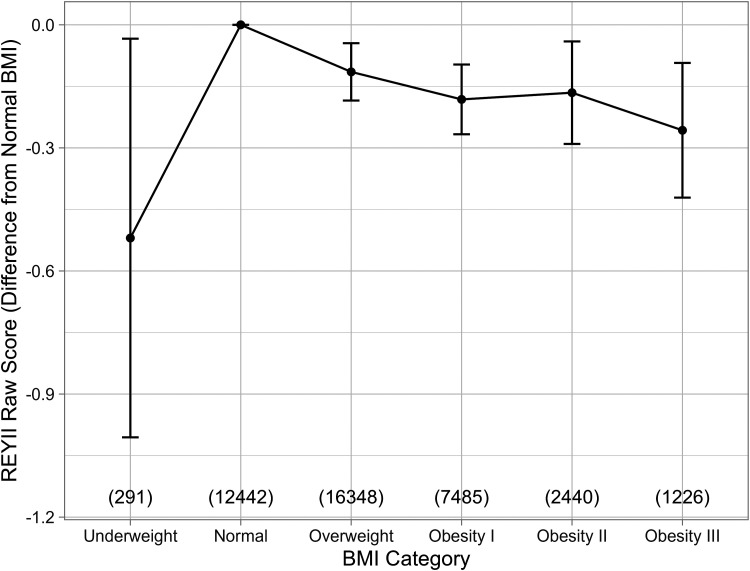
Contrast estimates and 95% confidence intervals of BMI categories (as compared to normal BMI) in linear regression model for REYII score. Participants with normal BMI had significantly better performance than all other BMI categories. The number of participants in each BMI category is provided in parentheses.

The mean raw REYII scores for each BMI category are provided in Supplemental Figure 1, indicating that normal BMI was associated with the best memory performance. A linear regression model was fit with the REYII score as the outcome, and all explanatory variables and interactions listed in Table 2 were included in the model. Table 2 also reports the estimated effect size (partial eta-squared), which measures the proportion of variance in REYII score attributable to each variable, after accounting for all other variables in the model. BMI classification was found to have a weak (η^2^ = 0.8%) effect on memory performance, with less than 1% of REYII score variability being explained by BMI classification, after accounting for other variables. The model's parameter estimates and 95% confidence intervals are reported in Supplemental Table 2. Figure 1 shows the estimated contrasts for BMI classification, demonstrating an inverted U relationship where normal BMI was associated with better REYII performance compared to underweight BMI, overweight, and obese I, II, and III BMI. The largest effects on REYII performance came from age (η^2^ = 6.9%) and sex (η^2^ = 5.4%), with older participants and males performing significantly worse overall. These effects are evident in the plot of the mean REYII score by age and sex, as shown in Supplemental Figure 2.

**Table 2.** Terms included in the final linear regression model for REYII score, including their effect sizes (Type III partial eta-squared) and 95% confidence intervals.

Term	Partial η^2^	95% C.I. for partial η^2^
(Intercept)	N/A	N/A
BMI Classification	0.0080	[0.0063, 0.0097]
Age Group	0.0688	[0.0641, 0.0735]
Sex	0.0543	[0.0502, 0.0587]
Education	0.0270	[0.0240, 0.0302]
Geostrata	0.0101	[0.0078, 0.0116]
Cohort	0.0023	[0.0014, 0.0033]
Race	0.0025	[0.0016, 0.0036]
Age * Sex interaction	0.0005	[0.0001, 0.0009]
Age * Cohort interaction	0.0028	[0.0018, 0.0038]
Sex * Cohort interaction	0.0016	[0.0009, 0.0025]
Education * Cohort interaction	0.0010	[0.0004, 0.0016]

### Association between BMI classification and MCI

**Figure 2.**
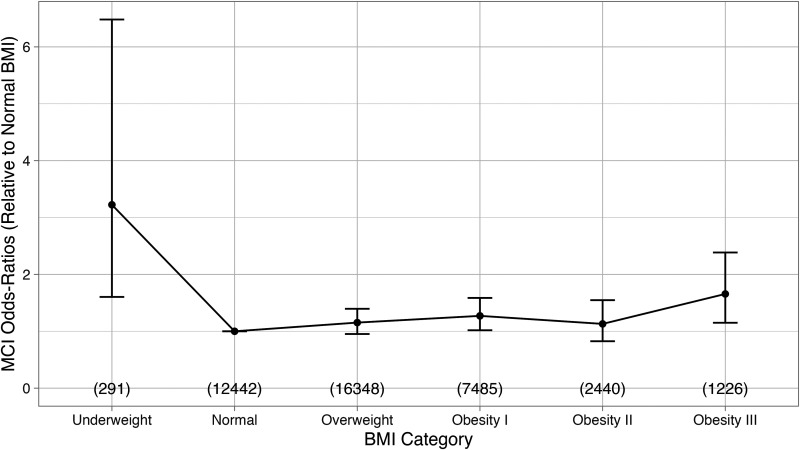
Odds Ratio estimates and 95% confidence intervals of BMI categories (as compared to normal BMI) in logistic regression model for MCI risk. The number of participants in each BMI category is provided in parentheses.

The absolute MCI rates for each BMI category are provided in Supplemental Figure 3, where the rate of MCI for those with low BMI/ underweight (20%) was 5 times as high as for those with normal BMI (4%). A logistic regression model was fit with MCI status as the outcome, and all explanatory variables and interactions listed in Supplemental Table 3 included in the model. Supplemental Table 3 also reports the odds ratio and 95% confidence intervals associated with the levels of every term. Figure 2 shows the estimated odds ratios by BMI classification, showing that normal BMI was associated with the lowest risk of MCI. Being underweight was associated with the highest increase in MCI risk of 3.22 (95% CI [1.627, 6.391]) times the normal BMI rate. The effects for overweight and obesity classes I and II were less pronounced, except for obesity class III which resulted in an increase of 1.65 (95% CI [1.158, 2.368]) times the normal BMI rate. In terms of other variables, the rate of MCI decreased monotonically with age for males, but was more stable with respect to age for females. This interaction of age with sex is evident in Supplemental Figure 4, showing estimates of MCI rates by these two variables.

### Association between 3-year changes in BMI and changes in memory performance

Table 3 shows that for all BMI change categories, the vast majority (range 89.9 to 90.9%) had no change in memory performance. Based on our chi-square test analysis, there was no meaningful association between change in BMI and change in memory performance (Pearson chi-square statistic =8.57, p = 0.38, Cramér's V = 0.01).

**Table 3.** Most participants demonstrated no change in memory performance between baseline and follow-up 1 (approximately 3 years). There was no significant association between change in BMI and change in memory performance.

	BMI no change^a^	BMI decreased >10%	BMI decreased 5–10%	BMI increased 5–10%	BMI increased >10%
No change in memory performance	89.9%	90.2%	90.0%	90.3%	90.9%
Decline in memory performance	4.39%	4.4%	5.1%	4.2%	3.8%
Improvement in memory performance	5.74%	5.4%	4.9%	5.5%	5.3%

^a^ For all cells, percentage of sample reported.

## Discussion

This study aimed to investigate the relationship between BMI and cognitive function among older Canadian adults using the CLSA database. In line with our hypothesis, this study found evidence for an inverted-U relationship between cognitive function and BMI, with the underweight class having the worst memory performance compared to the overweight and obese BMI categories. Our results suggest that having a normal BMI is associated with optimal cognitive performance and is in line with other studies that have found an inverted U relationship between BMI and cognition.^16^ Evidence for the obesity paradox (higher BMI being associated with better cognitive performance) was not found in our study and could be due to the younger age range of participants (age 45 plus) rather than age 75 and over where this phenomenon has been demonstrated more reliably.^17^

There are several possible mechanisms linking normal BMI with optimal cognitive performance. One possible mechanism may involve leptin, a hormone secreted by adipose tissue that aids regulation of appetite and satiety. Research has shown that among those with normal BMI, higher levels of leptin are associated with better cognitive performance.^30^ In animal models, leptin has been shown to slow neurodegeneration, increase beta amyloid clearance, improve hippocampus neuron survival and promote synaptic plasticity.^31^ A recent meta-analysis also found lower leptin levels among those with AD compared to cognitively normal participants.^32^ Obesity on the other hand is linked to systemic inflammation,^4,31^ and one possible outcome of increased inflammatory biomarkers markers is leptin resistance.^31^ Other potential mechanisms that link obesity with poor cognitive function include insulin resistance^33,34^ and the inflammatory C-reactive protein (CRP).^34,35^ Inflammation also may lead to impaired endothelial function and vascular blood flow which can then lead to white matter hyperintensities and reduced brain volume^35,36^ which are associated with cognitive dysfunction.

Surprisingly, we found that the negative effects of low BMI/ being underweight on cognition were more pronounced than being overweight and obese. In our final model accounting for all confounders, being underweight was associated with 3.22 times greater odds of meeting criteria for MCI compared to normal BMI. Possible mechanisms linking low BMI to worse cognitive function include lower production of leptin,^37^ cerebrovascular pathology,^38^ and higher levels of inflammatory markers.^39^ Low BMI can also be a result of other underlying health conditions and nutritional deficiencies. Studies have found that nutritional risk (i.e., malnutrition and risky food intake) is associated with poorer cognitive function for older adults and is an area that warrants further investigation.^40,41^ Further study is also needed with regards to the factors mediating the relationship between low BMI and cognitive function and how they can be targeted using novel therapeutic interventions to prevent the detrimental effects of low BMI on cognition.

We found that the association between BMI and memory performance did not differ according to sex, age and race (i.e., there were no significant interactions between BMI and any of these demographic variables). This finding is contrary to previous reports that point to differences in the relationship between BMI and cognition according to sex,^11,12^ age,^9^ and race^42–44^ and may be due to the larger variability among our sample, particularly with regards to age, race and BMI, that may be masking any significant interactions. One interesting finding from our study that warrants further investigation was the worse memory performance and higher odds of MCI among the non-White compared to White participants. We note, however, that the number of participants within the non-White category was small (4% of sample) which is why we had to group all racial minority groups into one category. Nonetheless, we suggest further investigation into why cognitive performance among the non-White participants was worse (e.g., due to language and cultural differences) and the potential impact of social determinants of health on cognitive function among older adults.

We found no significant associations between changes in BMI and changes in memory performance after 3 years. This lack of association may be due to the short follow-up window of 3 years which may not have allowed for many participants to demonstrate significant changes in both variables. Indeed, approximately 90% of participants showed no change in cognitive performance at follow-up. Some previous studies have reported associations between BMI changes and rates of cognitive decline over 3 years which may be due to differences in sample characteristics, measures, and criteria used^17,45,46^ compared to our study. It should also be noted that memory performance, as measured by the REYII, is subject to natural and measurement variability across testing sessions (e.g., due to factors such as sleep quality), which could lead to regression to the mean and erode the power of this test and thus explaining why our results were nonsignificant; BMI, by contrast, is less prone to such variability. Overall, we suggest further examination using longer follow-up data now available from the CLSA to determine whether longitudinal changes in cognition are associated with changes in BMI.

There are several limitations to our study that should be noted. Objective 1 of our study incorporated a cross-sectional study design, and so causal inferences cannot be made such as whether cognitive decline preceded weight loss or weight loss led to cognitive decline. The relationship is likely bidirectional as has been found with regards to the association between adiposity and executive function.^47^ The use of BMI as our measure of obesity is also a limitation as BMI does not differentiate between muscle mass and fat mass. Measures such as waist circumference, waist-to-hip ratio, and use of scans such as Dual-Energy X-ray Absorptiometry (DEXA or DXA) that provide measures of visceral adiposity may be more useful and should be prioritized in future studies. However, it has been recommended for clinicians and researchers to take a more holistic approach when defining obesity.^48^ We also note that there were differences between the Tracking and Comprehensive cohort with regards to how BMI was reported. For the Tracking cohort, BMI was calculated based on self-reported height and weight. For the Comprehensive cohort, BMI was physically measured in person. For both cohorts, severe cognitive impairment limiting ability to understand purpose of the study (and provide informed consent) and/or provide reliable data was an exclusion criteria that could have led to selection bias of participants with more intact cognition than the rest of the Canadian population. Another limitation is that the definition of MCI we used has not been formally validated and we recommend that an MCI variable be added/collected as part of the CLSA. Finally, the low BMI/underweight category made up only 0.7% of the total sample (291 participants). The small number of participants in this category, although accounted for in the analyses, may still have partially skewed results. Further study to determine whether our findings are replicated in larger underweight samples is therefore needed. There was also a very small proportion of participants who were non-White (<5%) within the sample, which may reflect selection bias given that the CLSA was conducted only in English or French, limiting generalizability of the results to the rest of the Canadian population.

### Conclusions

This study examined the relationship between the range of BMI classifications and presence of MCI and memory performance and amongst older Canadian adults, the first of its kind among a large Canadian sample. We were able to replicate an inverted-U relationship between cognitive function and BMI in a large Canadian sample but the detrimental effects of being underweight were much more pronounced than being obese. These results suggest the potential importance of developing strategies to prevent low BMI among older adults, such as nutritional and exercise programs, medication management and oral health programs, as they may help support cognitive health. We discussed possible pathways that explain our results; however, further study to elucidate the mechanisms mediating the relationship between low BMI and cognitive function is also needed (e.g., nutritional risk, markers of inflammation, brain volume, and vascular risk factors). Finally, our study did not find any significant associations between changes in BMI with changes in memory performance after 3-year follow up, however, we recommend a further longitudinal follow-up study using more recent data available from the CLSA.

## Supplemental Material

sj-docx-1-alz-10.1177_13872877261464179 - Supplemental material for Low body mass index is associated with higher rates of mild cognitive impairment and worse memory performance among older Canadian adults: An analysis of the Canadian Longitudinal Study on Aging (CLSA) database Supplemental material, sj-docx-1-alz-10.1177_13872877261464179 for Low body mass index is associated with higher rates of mild cognitive impairment and worse memory performance among older Canadian adults: An analysis of the Canadian Longitudinal Study on Aging (CLSA) database by Nadine Akbar, Dewei Lin, Yan Hoi Caleb Cheung, Asha Mohamed, Mohammad Chowdhury and Sotirios Damouras in Journal of Alzheimer's Disease
