# Supplementary material for: Low body mass index is associated with higher rates of mild cognitive impairment and worse memory performance among older Canadian adults: An analysis of the Canadian Longitudinal Study on Aging (CLSA) database
Source: J Alzheimers Dis. 2026 Jul 11;112(4):1945–54. doi: 10.1177/13872877261464179 (PMC13433890; doi:10.1177/13872877261464179)
Supplement: Supplementary file 1 [file sj-docx-1-alz-10.1177_13872877261464179.docx]

**Supplemental Material**

**Low body mass index is associated with higher rates of mild cognitive impairment and worse memory performance among older Canadian adults: An analysis of the Canadian Longitudinal Study on Aging (CLSA) database**

**Supplemental Table 1.** Data exclusion table for Comprehensive and Tracking datasets at baseline and 3-year follow-up.

|  | Baseline | | | 3-year follow-up | | | Combined baseline and follow-up |
| --- | --- | --- | --- | --- | --- | --- | --- |
|  | Comprehensive | Tracking | Total | Comprehensive | Tracking | Total | Total |
| Total before exclusion | 30097 | 21241 | 51338 | 27768 | 17050 | 44818 | 44815 |
| Total number of participants uniquely excluded | 4988 | 6118 | 11106 | 8875 | 5038 | 13913 | 17034 |
| Reasons for exclusion |  |  |  |  |  |  |  |
| Missing Data | 3331 | 5215 | 8546 | 7257 | 3646 | 10903 | 13971 |
| Pregnant | <15 | <15 | <15 | <15 | <15 | <15 | <15 |
| Stroke or CVA | 582 | 413 | 995 | 1594 | 409 | 2003 | 2193 |
| Mini-stroke or TIA | 1146 | 812 | 1958 | 2208 | 750 | 2958 | 3345 |
| Parkinsonism | 139 | 89 | 228 | 1196 | 100 | 1296 | 1315 |
| Multiple Sclerosis | 217 | 154 | 371 | 1200 | 122 | 1322 | 1370 |
| Epilepsy | 344 | 181 | 525 | 1302 | 287 | 1589 | 1655 |
| Dementia or Alzheimer’s Disease | 68 | 43 | 111 | 73 | 52 | 125 | 165 |
| Total after exclusion | 25109 | 15123 | 40232^a^ | 12012 | 12012 | 30905 | 27781^b^ |

^a^Sample used for Objective 1

^b^Sample used for Objective 2

**Supplemental Table 2.** Terms included in the final linear regression model for REYII score, including their parameter estimates (contrasts) and associated 95% confidence intervals.

| **Term** | **Estimate** | **95% CI** |
| --- | --- | --- |
| (Intercept) | 4.691 | [4.505, 4.876] |
| BMI Classification [Underweight] | -0.520 | [-0.996, -0.044] |
| BMI Classification [Overweight] | -0.115 | [-0.183, -0.046] |
| BMI Classification [Obesity I] | -0.182 | [-0.265, -0.099] |
| BMI Classification [Obesity II] | -0.165 | [-0.288, -0.043] |
| BMI Classification [Obesity III] | -0.257 | [-0.418, -0.096] |
| Age [>55-65] | -0.424 | [-0.528, -0.320] |
| Age [>65-75] | -0.987 | [-1.109, -0.866] |
| Age [>75-85] | -1.881 | [-2.017, -1.744] |
| Sex [M] | -0.798 | [-0.902, -0.693] |
| Education [Medium] | 0.446 | [0.291, 0.600] |
| Education [High-lower] | 0.629 | [0.465, 0.793] |
| Education [High-upper] | 0.833 | [0.702, 0.965] |
| Geostrata [AB _Calgary] | 0.024 | [-0.129, 0.177] |
| Geostrata [AB _Non_DCS] | -0.226 | [-0.452, 0.001] |
| Geostrata [BC _Non_DCS] | -0.149 | [-0.390, 0.092] |
| Geostrata [BC _SFU_UBC] | -0.057 | [-0.210, 0.095] |
| Geostrata [BC _Victoria] | 0.153 | [0.018, 0.288] |
| Geostrata [MB _Manitoba DCS] | -0.069 | [-0.207, 0.070] |
| Geostrata [MB _Non_DCS] | -0.198 | [-0.472, 0.075] |
| Geostrata [NB _Non_DCS] | -0.389 | [-0.653, -0.126] |
| Geostrata [NL _Memorial] | -0.269 | [-0.424, -0.114] |
| Geostrata [NL _Non_DCS] | -0.324 | [-0.621, -0.027] |
| Geostrata [NS _Dalhousie] | -0.318 | [-0.451, -0.185] |
| Geostrata [NS _Non_DCS] | -0.402 | [-0.652, -0.152] |
| Geostrata [ON _Hamilton] | -0.554 | [-0.701, -0.407] |
| Geostrata [ON _Non_DCS] | -0.243 | [-0.431, -0.054] |
| Geostrata [PEI_Non_DCS] | -0.345 | [-0.591, -0.099] |
| Geostrata [QC _McGill] | -0.294 | [-0.434, -0.154] |
| Geostrata [QC _Non_DCS] | -0.190 | [-0.419, 0.038] |
| Geostrata [QC _Sherbrooke] | -0.399 | [-0.530, -0.269] |
| Geostrata [SK _Non_DCS] | -0.335 | [-0.577, -0.093] |
| Cohort [Tracking] | 0.044 | [-0.229, 0.317] |
| Race [non-White] | -0.541 | [-0.680, -0.402] |
| Age[>55-65] * Sex [M] interaction | -0.165 | [-0.298, -0.032] |
| Age[>65-75] * Sex [M] interaction | -0.240 | [-0.395, -0.085] |
| Age [>75-85] * Sex [M] interaction | -0.094 | [-0.259, 0.070] |
| Age [>55-65] * Cohort [Tracking] interaction | 0.350 | [0.205, 0.495] |
| Age [>65-75] * Cohort [Tracking] interaction | 0.606 | [0.432, 0.779] |
| Age [>75-85] * Cohort [Tracking] interaction | 0.615 | [0.422, 0.807] |
| Sex [M] * Cohort [Tracking] interaction | -0.346 | [-0.467, -0.226] |
| Education [Medium] * Cohort [Tracking] interaction | 0.174 | [-0.076, 0.423] |
| Education [High-lower] * Cohort [Tracking] interaction | 0.492 | [0.217, 0.767] |
| Education [High-upper] * Cohort [Tracking] interaction | 0.306 | [0.094, 0.519] |

**Supplemental Table 3.** Terms included in the final logistic regression model for MCI risk, including their estimated odds ratios and associated 95% confidence intervals.

| **Term** | **Odds Ratio** | **95% CI** |
| --- | --- | --- |
| (Intercept) | 0.025 | [0.014, 0.044] |
| BMI Classification [Underweight] | 3.224 | [1.627, 6.391] |
| BMI Classification [Overweight] | 1.153 | [0.956, 1.390] |
| BMI Classification [Obesity I] | 1.271 | [1.023, 1.580] |
| BMI Classification [Obesity II] | 1.130 | [0.830, 1.538] |
| BMI Classification [Obesity III] | 1.656 | [1.158, 2.368] |
| Age [>55-65] | 0.577 | [0.447, 0.745] |
| Age [>65-75] | 0.729 | [0.537, 0.989] |
| Age [>75-85] | 0.587 | [0.389, 0.884] |
| Sex [M] | 1.001 | [0.797, 1.256] |
| Education [Medium] | 1.362 | [0.963, 1.927] |
| Education [High-lower] | 1.222 | [0.836, 1.787] |
| Education [High-upper] | 1.303 | [0.960, 1.769] |
| Geostrata [AB _Calgary] | 0.971 | [0.569, 1.657] |
| Geostrata [AB _Non_DCS] | 0.671 | [0.335, 1.344] |
| Geostrata [BC _Non_DCS] | 0.837 | [0.405, 1.730] |
| Geostrata [BC _SFU_UBC] | 1.160 | [0.731, 1.841] |
| Geostrata [BC _Victoria] | 0.708 | [0.439, 1.143] |
| Geostrata [MB _Manitoba DCS] | 1.542 | [0.960, 2.477] |
| Geostrata [MB _Non_DCS] | 1.109 | [0.504, 2.439] |
| Geostrata [NB _Non_DCS] | 1.606 | [0.743, 3.472] |
| Geostrata [NL _Memorial] | 1.493 | [0.941, 2.369] |
| Geostrata [NL _Non_DCS] | 2.486 | [1.196, 5.170] |
| Geostrata [NS _Dalhousie] | 1.948 | [1.267, 2.994] |
| Geostrata [NS _Non_DCS] | 1.825 | [0.884, 3.768] |
| Geostrata [ON _Hamilton] | 1.570 | [1.014, 2.429] |
| Geostrata [ON _Non_DCS] | 1.380 | [0.763, 2.496] |
| Geostrata [PEI_Non_DCS] | 1.733 | [0.834, 3.603] |
| Geostrata [QC _McGill] | 1.706 | [1.070, 2.719] |
| Geostrata [QC _Non_DCS] | 1.244 | [0.631, 2.451] |
| Geostrata [QC _Sherbrooke] | 1.559 | [1.012, 2.403] |
| Geostrata [SK _Non_DCS] | 1.663 | [0.833, 3.322] |
| Cohort [Tracking] | 0.929 | [0.638, 1.352] |
| Race [non-White] | 4.657 | [3.694, 5.871] |
| Age [>55-65] * Sex [M] interaction | 1.172 | [0.847, 1.621] |
| Age [>65-75] * Sex [M] interaction | 0.530 | [0.363, 0.774] |
| Age [>75-85] * Sex [M] interaction | 0.419 | [0.245, 0.718] |
| Age [>55-65] * Cohort [Tracking] interaction | 1.207 | [0.857, 1.699] |
| Age [>65-75] * Cohort [Tracking] interaction | 1.309 | [0.877, 1.954] |
| Age [>75-85] * Cohort [Tracking] interaction | 2.157 | [1.286, 3.619] |

**Supplemental Figure 1.** Estimated mean REYII raw score and associated 95% confidence interval for each BMI category; sample sizes are provided in parentheses.

**Supplemental Figure 2.** Estimated mean REYII raw score and associated 95% confidence interval by age group and sex; age group sample sizes are in parentheses.

**Supplemental Figure 3.** Estimated absolute MCI rates and approximate 95% confidence intervals for each BMI category; sample sizes in parentheses.

**Supplemental Figure 4.** Estimated absolute MCI rates and approximate 95% confidence intervals by age group and sex; age group sample sizes in parentheses.
